# Robustness of the self-referential process under normobaric hypoxia: an fNIRS study using the GLM and homologous cortical functional connectivity analyses

**DOI:** 10.3389/fnhum.2024.1337798

**Published:** 2024-03-12

**Authors:** Takehiro Minamoto, Naoaki Kawakami, Takehiko Tsujimoto

**Affiliations:** ^1^Department of Health and Behavioral Sciences, Graduate School of Human and Social Sciences, Shimane University, Matsue, Japan; ^2^Faculty of Human Sciences, University of Tsukuba, Tsukuba, Japan

**Keywords:** self-referential effect, hypoxia, fNIRS, default mode network, coherence analysis

## Abstract

**Introduction:**

Hypoxia has been reported to impair psychological functions, such as working memory and decision-making. However, few studies have examined hypoxia’s effect on social cognition.

**Methods:**

Using a self-referential task, the present study investigated normobaric hypoxia’s effect on the self-referential process. Additionally, we measured brain activity during the task with fNIRS and performed conventional univariate analysis with the general linear model (GLM) as well as homologous cortical functional connectivity analysis.

**Results:**

The results revealed that normobaric hypoxia impaired recognition of adjectives in the other-reference condition but not in the self-reference. The GLM analysis did not detect differences in brain activity between the self- and other-reference conditions, suggesting that GLM analysis may not be suitable for examining self- and other-reference conditions’ neural correlates. The homologous cortical connectivity analysis revealed that the connectivity’s magnitude was greater in the self-reference than in the other-reference conditions in the normoxic group. However, such a decrease in connectivity in the other-reference conditions was not observed in the hypoxic group, possibly to compensate for cognitive decline induced by the hypoxia.

**Conclusion:**

Considering that homologous connectivity reflects the default mode network, which is supposedly linked to continuous self-reference, stable strength of the connectivity in the self-reference condition under the hypoxia may suggest robust nature of the self-reference process under normobaric hypoxia.

## Highlights

• The self-referential paradigm showed that the self-referential process was reserved under normobaric hypoxia, but the other-referential one was not.
• Brain activity was similar between self- and other-referential processes, irrespective of oxygenic level in the univariate analysis.
• The homologous cortical functional connectivity, which is linked to DMN activity, was greater in the self-reference than in the other reference condition in the normoxic group, whereas such a difference was absent in the hypoxic group.

## Introduction

At higher altitudes, we experience psychological dysfunctions in working memory, spatial orientation, and decision-making, among others. These dysfunctions are primarily attributable to a lack of oxygen to the central nervous system, so-called hypoxia. In a recent systematic review, McMorris et al. (2017) reported that acute hypoxia negatively impacts a wide range of cognitive functions—including central executive as well as non-executive perceptual functions. Their findings were interesting in that the negative impact on central executive functions was similar to that on non-executive functions, which was counterintuitive to their initial prediction.

Studies have hypothesized that acute hypoxia reduces higher-order cognitive functions by affecting the prefrontal cortex (PFC). For instance, Davranche et al. (2016) measured the PFC’s activity while participants performed the Simon task, which produces cognitive conflict, at an altitude of 4350 m. They found that the tissue oxygenation index (TOI)—computed using the NIRS signal’s slope—increased at the high altitude. As task performance was undermined at the high altitude, the increase in PFC activity was interpreted as a greater demand for neural resources, which were insufficient to compensate for the cognitive decline observed in the experiment. In another fNIRS study, acute hypoxia decreased the PFC’s activation under cognitive conflict (Ochi et al., 2018). In their preliminary study, oxy-Hb signals were measured while participants performed part of the Stroop task under normobaric hypoxia (13.5% O_2_ and 0.03% CO_2_ in nitrogen N_2_; equivalent altitude of approximately 3,500 m). Although the magnitude of Stroop interference did not differ between the hypoxic and normoxic conditions, the left dorsolateral PFC’s interference-related activity was greater in the normoxic than in the hypoxic condition. Owing to the paucity of research in this area, the direction of the hypoxic effect on the PFC was inconsistent; however, acute hypoxia seemingly affects the PFC’s conflict-related activity.

### Hypoxia and self- and other-recognition

While studies have examined hypoxia’s effects on perception, attention, short-term and working memory, and decision-making, other psychological functions have remained uninvestigated with respect to their vulnerability to hypoxia. For instance, a previous systematic review of acute hypoxic effects did not include any long-term memory tasks (McMorris et al., 2017), and the authors emphasized the need for research on long-term memory. Furthermore, a hypoxic effect on social cognition has received far less attention. In studies on social cognition, the concept of the self has been extensively explored in philosophy, psychology, and cognitive neuroscience. The self has been proposed to comprise multiple concepts—namely, the proto-self, mental self, and autobiographical or narrative self (Northoff et al., 2011). The proto-self refers to the sensorimotor and affective perception of one’s own body (Panksepp, 1998; Damasio, 1999). The mental self refers to the cognitive content associated with the self, such as one’s name or house (Damasio, 1999, 2003). Finally, the autobiographical or narrative self refers to the self-representation built on one’s own autobiographical memory, such as the recognition of one’s own personality traits (Damasio, 1999; Gallagher, 2000).

These concepts related to the self are always in one’s awareness and constantly updated and, hence, seem to resist internal and external disturbances. In fact, neuropathologies of the self—such as delusional misidentification syndrome and nurturing syndrome—require multilevel deficits to emerge, according to Feinberg’s four-tiered model (Feinberg, 2011). Feinberg (2011) proposed that interactions in three lower levels—namely, cognitive deficits (level 1), self-related deficits (level 2), and defense/adaptation/motivation (level 3)—create self-pathology syndromes at the fourth level. Specifically, deficits in a single or several cognitive functions are insufficient to precipitate neuropathologies of the self. On the contrary, non-self-processing, such as memory retrieval of semantic knowledge, is likely to be easily exacerbated because the processing depends predominantly on the cognitive systems at level 1.

### Self-recognition and default mode network

Due to its engineering principle, generally, fNIRS has been used to measure brain activity in lateral cortical regions (Villringer et al., 1993), but its application to the study of psychological processing in medial cortical regions has been rare. This issue falls into self-referential processing, which relies on the cortical midline structures (Northoff et al., 2006). However, recent studies have begun capturing neural responses that may be associated with medial cortical regions using multivariate analyses (Sasai et al., 2011). Specifically, the authors measured resting state brain activity using fNIRS, and analyzed neural coherences between homologous contra-lateral regions and those between fronto-posterior regions in low-frequency band (LF: 0.06–0.08 Hz) and very low frequency band (VLF: 0.009–0.02 Hz). The results showed a significant interaction indicating that magnitude of the VLF coherence between homologous regions was far greater than that of the VLF coherence between the fronto-posterior regions. By contrast, in the LF band, difference in magnitude of coherence was reduced between the homologous and fronto-posterior regions, which was primarily due to transient increase in the LF coherence in the fronto-posterior regions. Those results seem to indicate that the LF coherence reflects transient neural coordination across distinct brain regions to cope with a single event such as stimulus-processing, as discussed in Sasai et al. (2011). Meanwhile, the VLF homologous coherence seems to be closely tied with continuous spontaneous neural coordination across distinct brain regions during resting state.

In the present study, we propose a primitive exploratory hypothesis that the VLF coherence between the contra-lateral homologous regions reflects some aspects of the default mode network (DMN), which comprises the medial prefrontal cortex, orbital frontal cortex, lateral temporal cortex, inferior parietal lobe, posterior cingulate cortex, hippocampus, and parahippocampal cortex (Raichle, 2015). The hypothesis resides in the findings where grater activation of the DMN regions is repetitively reported during a resting state (Raichle et al., 2001; Buckner and Vincent, 2007; Qin and Northoff, 2011). We surmise that the VLF homologous coherence reflects some property of the medial cortical regions, because neural activities of the superficial lateral regions are measurable by fNIRS. More specifically, if conventional univariate analysis cannot detect any activity of the lateral DMN region during a task that requires activity of the DMN regions, but the homologous connectivity analysis finds some differences during the task, the result may indicate differences in the non-lateral regions, including the medial cortical regions.

The self-referential task, which requires continuous processing of self-referential stimuli, has been shown to activate DMN regions (Northoff and Bermpohl, 2004), including the medical prefrontal cortex. More recently, the ventromedial prefrontal cortex (vmPFC), which is one of the core regions of the DMN, has been shown to have two distinctive functions by exerting different brain regions (Sui and Humphreys, 2017). The one is self-anchor function such as internally guided decision making (e.g., subjective preference, or beliefs). The vmPFC is repeatedly reported to be activated while participants made decision based on their subjective criterion, coupling with the perigenual anterior cingulate cortex, posterior cingulate cortex, and superior temporal gyrus. The other function is self-binding, which plays an important role when we perceive self-related information in the environment. For instance, the vmPFC strongly activated when a geometrical shape paired with self was presented relative to those paired with a friend or stranger (Sui et al., 2013). Interestingly, the vmPFC showed greater coupling with the left posterior superior temporal sulcus (LpSTS) when geometry shapes paired with self were presented. The result indicates that neural coupling between the vmPFC and LpSTS improves perceptual integration, prioritizing self-related information (Sui and Humphreys, 2015). In sum, the present study explores the primitive hypothesis that the non-conventional multivariate functional connectivity analysis can detect brain activity potentially associated with the medial cortical regions associated with the DMN.

### Aims and hypotheses of the present study

The present study investigated how normobaric hypoxia affects self- and other-referential processing. Using the self-referential task, participants were asked to rate how personality-related adjectives matched themselves (self) and the previous Japanese prime minister (other). Considering that hypoxia is associated with the disturbance of varied fundamental cognitive functions (i.e., level 1 of the four-tiered model), it is reasonable to hypothesize that acute hypoxia selectively impairs the other-referential process while reserves the self-referential one, because these disturbances are insufficient to impair self-related processes. Additionally, we measured brain activity during self- and other-referential processes using functional fNIRS. As it has been repeatedly reported that the self-referential task relies on the cortical midline structures, and that fNIRS measures superficial cortical regions’ neural activity, it is anticipated that conventional univariate analysis fails to detect differences in brain activity between self- and other-referential processes under acute hypoxia. Importantly, homologous connectivity in the VLF band has been found to increase during resting state, as stated above, and similar brain activity has been repeatedly observed during resting state and when performing self-referential tasks (Qin and Northoff, 2011). Considering these similarities, it can be hypothesized that acute hypoxia affects homologous cortical connectivity in the VLF band, resulting in poorer referential processes pertaining to other individuals. The VLF homologous connectivity is predicted to be reserved during self-reference under hypoxic conditions because of its robust nature.

## Materials and methods

### Participants

Participants were graduate and undergraduate students enrolled in Shimane University. Because participants were required to have high Japanese proficiency, only Japanese students were recruited. Overall, 93 participants (55 women) participated in the experiment, and their mean age was 19.32 (*SD* = 2.04). The sample size was determined, referring to the systematic review literature with the meta-analytic regression analysis (McMorris et al., 2017). The participants provided written consent after agreeing to participate in the study protocol, which was thoroughly explained by an experimenter. Additionally, they completed a health condition check sheet (Supplementary Figure 1) and declared that they experienced no symptoms of the common cold and had no history of cardiovascular disease or altitude anoxia. Hence, previous medical conditions were not tested as for their effects on the brain responses. Before entering the hypoxic room, a heart rate monitor (H10, Polar, Finland) and pulse oximeter (ATP-W03, Fukuda Denshi, Japan) were attached to their chest and left index finger to monitor their heart rate and saturation of percutaneous oxygen (SpO_2_). We confirmed that their SpO_2_ scores were 96% or greater in a normoxic environment. Their participation was compensated by a 1000-yen gift card. The study protocol was approved by the Institutional Review Board of the Faculty of Human Sciences at Shimane University. No part of the study procedures and analyses was pre-registered prior to the research being conducted. Because the Institutional Review Board permitted the present study under the condition that the collected data are handled only in offline environment, we are not able to make those data available online.

### Apparatus

Cerebral blood flow was measured using OEG-17APD (Spectratech Inc., Tokyo, Japan). Using two wavelengths of near-infrared light (770 and 840 nm), the device captured oxy-Hb and deoxy-Hb signals. A pair of 2 × 3 sensor pallets was selected to cover the bilateral fronto-temporo-parietal regions, resulting in seven measurement channels in each hemisphere. The emitter and detector probes were distanced at 3 cm apart in each hemisphere. A light-shielded cloth was placed on the probes to prevent exposure to room light. The NIRS device was connected to a laptop PC (ThinkPad T550, NEC Lenovo Japan Group, Tokyo), and the OEG17Control software (Spectratech Inc., Tokyo) was installed to record continuous oxy- and deoxy-Hb signals. Another laptop PC (ThinkPad SL510, Lenovo [Singapore] Pte. Ltd., Singapore) was used for stimulus presentation, response retrieval, and trigger output to the NIRS device. Using an Express card (EC1PECPS, Ltd., London, Ontario), trigger outputs—synchronized with the experimental stimuli—were sent to the OEG-17APD. The card was attached to a parallel port connected to a customized BNC cable (Physio-Tech Co., Ltd., Tokyo). The BNC cable was plugged into the NIRS device’s back socket. A presentation software (Presentation 20.1; Neurobehavioral Systems, Inc., Berkeley, CA, USA) was used for stimulus presentation, response retrieval, and trigger generation. Behavioral responses were collected using a wired 10-key pad (NT-18USV; Sanwa Supply Inc., Okayama).

The hypoxic room had a volume of 37.5 m^3^ (length 3.0 m; width 5.0 m; height, 2.5 m) and was sealed except one ventilation opening. A control unit (YHS-415, YKS, Japan) generated air with a low oxygen concentration (i.e., increased nitrogen concentration) to achieve a set oxygen concentration (13.5% O_2_) in the room. Carbon dioxide concentration was constantly monitored and was confirmed to not exceed 2,000 ppm.

### Stimulus

Overall, 140 Japanese adjectives were retrieved from previous studies (Aoki, 1971), 90 and 50 of which were used for the self-referential task and as fillers in the subsequent word recognition task, respectively. Further, 90 words were assigned to one of three word-lists, and mean scores of word-frequency, social desirability, and character length were matched as follows: word-frequency (*M* = 4.89, *SD* = 1.06 for list A; *M* = 4.92, *SD* = 1.10 for list B; and *M* = 4.83, *SD* = 1.04 for list C), social desirability (*M* = 5.02, *SD* = 1.73 for list A; *M* = 5.03, *SD* = 1.72 for list B; and *M* = 5.04, *SD* = 1.71 for list C), character length (*M* = 4.13, *SD* = 0.97 for list A; *M* = 4.06, *SD* = 0.94 for list B; and *M* = 4.10, *SD* = 0.96 for list C). The values for the fillers were as follows: word frequency (*M* = 4.40, *SD* = 1.36), social desirability (*M* = 4.86, *SD* = 1.54), and character length (*M* = 4.08, *SD* = 1.05).

### Procedure

**FIGURE 1:**
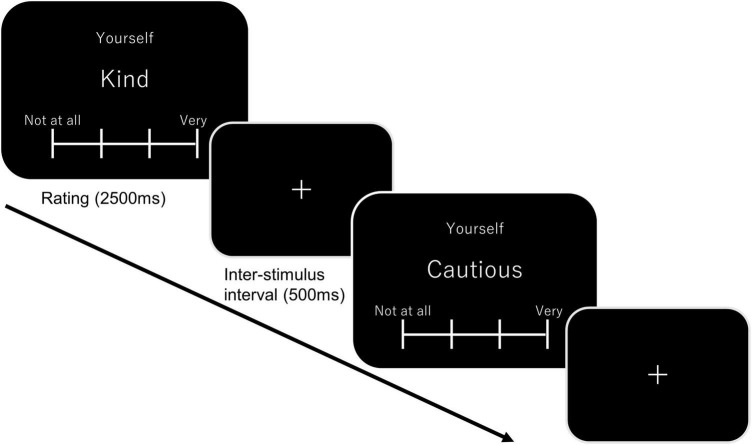
A schematic diagram of the self-reference task. In the self-block, “Yourself” was displayed on top of the screen below which showed a target adjective. Participants were required to judge how effectively the adjective described themselves. Each adjective was presented for 2500 ms, which was followed by an inter-stimulus interval (500 ms). A total of ten adjectives were presented in one block. In the other block, “Yourself” was replaced with “Mr. Abe” (the former Japanese Prime Minister). In the letter block, “Letters” was presented on top of the screen, and participants were required to count number of characters consisting an adjective.

The participants were assigned to either the hypoxic (*n* = 44, 25 females) or normoxic group (*n* = 49, 30 females). The assignments were performed in a single-blind manner. After participants entered the hypoxic room, sensor pallets were attached on their scalp surface, covering the bilateral fronto-temporo-parietal regions. The top-central probes in each pallet were located at the C3 and C4 sites of the international 10–20 system. The pallets were fixed on the scalp using head and cheek bands. The participants were seated at rest for approximately 15 min, including the NIRS preparation, which was required to sufficiently decrease the SPO_2_ levels in the hypoxic group. In the self-referential task, participants were instructed to view adjectives presented on the PC monitor and make a judgment according to one of the three conditions. In the self-reference condition, participants were required to judge how well an adjective described themselves. They were forced to select one of four options—namely, not matching at all, not matching well, matching relatively well, and matching fairly well. The tenkey was rotated 90°counterclockwise, and each option was assigned to the 8-, 5-, 2-, and 00-key. In the other-reference condition, the participants were required to think about how well a presented adjective described the previous Japanese prime minister (Mr. Abe) with the same choices as given above. In the letter condition, participants were required to count the number of characters that comprised adjectives and report based on one of the following choices: 3, 4, 5, or more than 6. A block design was used in the present study, and 10 adjectives were presented in each block. As presented in Figure 1, the ongoing condition was presented on top of the screen, and the adjectives below were alternated every 3000 ms, including a 500 ms inter-stimulus interval. Each condition (i.e., self-reference, other-reference, and letter) was administered three times in a pseudorandomized order. The remaining blocks (30 s) were given between the blocks, as well as at the task’s beginning and end. To eliminate the effect of the word stimulus, we prepared three word lists (30 adjectives in each list) and assigned them to each condition in a counterbalanced manner across participants. The fNIRS pallets were removed after the self-referential task, and other cognitive tasks were performed for the subsequent 60 min. These results will be presented in a different paper.

The word recognition task was performed at the end of the experiment. Overall, 140 words were presented sequentially, and participants were required to make an old/new judgment—that is, whether they had observed the word in the self-referential task. Notably, 90 words were presented in the self-referential task, while the other 50 were new items. The tenkey was rotated 90°counterclockwise, and old and new responses were assigned to the 7-key and new to 1-key, respectively. If they provided an old response, they further reported their confidence level using the following three-point forced choice: high (7-key), low (4-key), or guess (1-key). Immediately after the recognition task, the participants left the hypoxic room, and their physical condition was examined before concluding the experiment.

### SpO_2_ data acquisition

SpO_2_ was continuously measured using a pulse oximeter at a sampling rate of 1 Hz throughout all phases of the experiment. To avoid interference with task performance, the probe was attached to the non-dominant index finger. To prevent the participants from knowing which group they were assigned to, masking was applied to the measuring device’s display.

### fNIRS data acquisition

Regional oxy- and deoxy-Hb signals, which were converted from optical densities, were measured during the self-referential task. The signals were collected at a sampling rate of 12.2 Hz. Cutaneous blood flow was removed online using the signal separation method (Yamada et al., 2012). Signal calibration was performed before performing the self-referential task. When signals did not fulfill the criterion, which ranged between 500 and 8000, failed probes were detached from the sensor pallets, and the experimenter moved the hairs near the probes and recalibrated them. As the study was approved under the condition that individual experiments should be completed within 2 h in a hypoxic environment, recalibration was performed twice at maximum and signal recording started even though all channels did not fulfill the criterion.

### SpO_2_ data analysis

Each participant’s SpO_2_ score was computed by averaging the values recorded during the task. As the normality assumption was confirmed by the Shapiro-Wilk test, group comparisons were performed using Welch’s *t*-test. Type-I error was defined as 0.05.

### Behavioral data analysis

Following previous studies, the self-referential effect was estimated based on performance on the recognition task (Rogers et al., 1977). The present study computed individual d’ in each experimental condition, by subtracting false alarm rate for the filler words from hit rates for each experimental words that had been presented in the self-referential task. Obtained d’s were analyzed using a two-way mixed ANOVA that included the oxygenic level (between factor) and task conditions (within factor). Shaffer’s modified sequentially rejective Bonferroni’s procedure was used for multiple comparisons. Type-I error was defined as 0.05. Reaction time was not analyzed because the participants were allowed to make self-paced responses that varied significantly across individuals.

### Univariate analysis for fNIRS data

A low-pass filter (1.0 Hz) was applied to the time-series data of the oxy- and deoxy-Hb signals using the OEG17Control software, and the filtered data were exported for subsequent analysis. We performed filtering and baseline correction using NIRS-SPM (Ye et al., 2009) running on MATLAB (Mathworks Inc., Natick, MA, USA). The initial time was established as the baseline, and a Gaussian filter was used as the low-pass filter. These data were detrended using a detrending method based on discrete cosine transform (DCT). Owing to numerous channels’ poor calibration (Supplementary Figures 2, 3), individual channels were visually inspected. For inspection, predicted oxy-Hb signals were computed by convolving the hemodynamic response function (HRF) and overlaid on a real signal. If a given channel’s real signal was excessively apart from the predicted signal or absent (Supplementary Figures 2, 3), the channel was marked as removed. Although the procedure suffers from statistical issues, where sample size varied across channels, it maintained sample size in some channels (e.g., *n*_*hypoxy*_ = 32 and *n*_*normoxy*_ = 38 in channel 16).

Brain activities in each block (i.e., self-reference, other-reference, and letter) and the rest block were estimated using a general linear model (GLM) running on MATLAB. When running the GLM, we firstly defined the hemodynamic brain function (HRF) using gamma functions. Thereafter, the HRF was convolved with boxcar functions, whereby boxcars were prepared (three blocks for each experimental condition and eight rest blocks) for a duration of 30 s. GLMs were performed individually to estimate the beta vectors of the three experimental and rest blocks as well as the error variance in each channel. For statistical analysis, the beta value of the rest block was extracted from those of the experimental blocks in individual participants, and two-way mixed ANOVAs were performed to examine the effects of the oxygenic level (between factor) and task condition (within factor). The false discovery rate (FDR) method was used to correct *p*-values for multiple comparisons (14 channels). Type-I error was defined as 0.05.

### Functional connectivity analysis for fNIRS data

Frequency-specific functional connectivity was computed using a method described in the previous study (Sasai et al., 2011). First, the three blocks’ time-series data were concatenated as single time-series data for each experimental condition (i.e., self-reference, other-reference, and letter-count). A bandpass filter ranging from 0.009 to 1.0 Hz was applied to each time-series data. The squared coherences were computed for each experimental condition and rest using Welch’s averaged modified periodogram method (512-point Fourier transform, Hamming window, and 256-point overlap). As homologous connectivity, which coheres with interhemispheric homologous regions, in a very low frequency range (VLF, 0.012–0.02 Hz) was demonstrated to be characteristic in the resting state (Sasai et al., 2011), we obtained the VLF in each condition for each participant. Additionally, the low frequency (LF) connectivity was extracted, which ranged from 0.06 to 0.08 Hz. As the LF range is in accordance with a typical hemodynamic response’s time scale (Sasai et al., 2011), we assumed LF connectivity as a proxy for event-related transient neural synchronization, rather than spontaneous synchronization. VLF and LF values were converted to z-scores using Fisher’s z-transformation prior to statistical analyses. A MATLAB signal processing toolbox (Mathworks Inc., Natick, MA, USA) was used to perform the connectivity analysis. As the present study was primarily focused on functional connectivity in the self- and other-reference conditions, we performed a two-way mixed ANOVA to examine the effects of oxygen level and reference condition on VLF and LF homologous connectivity. Type-I error was defined as 0.05.

## Results

### SpO_2_ results

**FIGURE 2:**
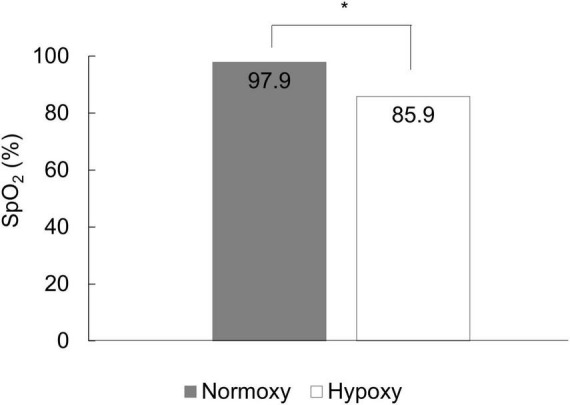
The mean SpO_2_ of the normoxic and hypoxic group. SpO_2_ in the hypoxic group was significantly lower than that in the normoxic group. *p < 0.05.

Data for 14 and 10 participants in the hypoxic and normoxic groups, respectively, could not be collected because of equipment problems during data collection. Therefore, 69 data points were analyzed. The SpO_2_ in the hypoxic and normoxic groups was 85.87 ± 0.41% and 97.85 ± 0.10%, respectively (Figure 2). Welch’s *t*-test revealed a significant difference between the groups (*t* = 28.341, df = 32.497, *p* < 0.001).

### Behavioral results

Data from one and two participant in the hypoxic and normoxic groups, respectively, were not included because of errors in data collection. Three participants in the hypoxic condition were excluded because one participant reported a sick feeling before the self-referential task and the other two exhibited deviated *d’* (i.e., greater than 2.5 *SD*s away from the overall mean in the self-reference condition [*n* = 1] or letter condition [*n* = 1]). Overall, 87 data points were analyzed.

**FIGURE 3:**
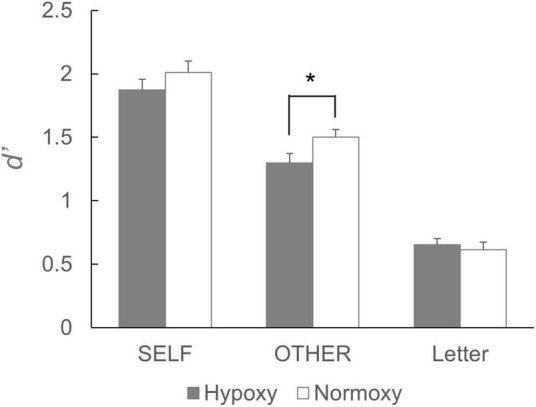
Performance of the subsequent recognition task. *d’* for the words that had been presented in the self-reference condition did not differ between the hypoxic and normoxic group. By contrast, *d’* for the words that had been presented in the other-reference condition was lower in the hypoxic group than the normoxic group. It should be noted that a statistical analysis yielded a trend toward significant interaction, and the following simple main effects analysis showed a significant main effect of the group in the other-reference condition. *p < 0.05.

The mean *d’*s are summarized in Figure 3. A robust self-referential effect was found in both hypoxic and normoxic groups; that is, d’ in the self-reference condition was greater than d’s in the other-reference and letter conditions. Further, performance in the other-reference condition was greater in the normoxic than in the hypoxic groups; however, such differences were not observed in the self-reference and letter conditions. A two-way mixed ANOVA revealed a significant main effect of the task condition, *F*_(2, 170)_ = 327.86, *p* < 0.001. Multiple comparison analyses revealed significant differences in all possible pairs (*p* < 0.001). The ANOVA indicated a trend toward significance in the interaction between oxygenic level and task condition, *F*_(2, 170)_ = 3.03, *p* = 0.051. A *post hoc* simple main effect analysis suggested a significant main effect of oxygen level in the other reference condition, *F*_(1, 85)_ = 4.44, *p* = 0.03. Such effects were observed neither in the self-reference condition, *F*_(1, 85)_ = 1.23, *p* = 0.27; nor in the letter condition, *F*_(1, 85)_ = 0.24, *p* = 0.62.

### fNIRS results

#### Univariate analysis

As stated in the section “Materials and methods,” the number of signal calibrations was limited to twice at maximum, which resulted in several channels’ exclusion from the data analysis. Individual participant data were excluded by visually inspecting the preprocessed oxy-Hb signals. Supplementary Table 1 (second column) lists the number of surviving samples from each channel. In channels 13 and 15, more than 30 samples fulfilled the inclusion criteria, while in channel 5 and 7, less than 10 samples did. Therefore, the present study analyzed each channel separately, and each channel had a different number of samples. This circumstance prevented us from drawing strong conclusions from the statistical analyses, especially for those with fewer than 20 samples in each group.

**FIGURE 4:**
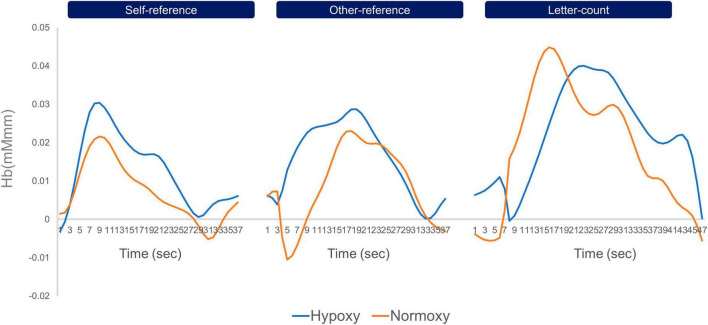
Timeseries oxy-Hb data of channel 16 averaged in the hypoxic and normoxic group. The left line graphs indicate timeseries change of oxy-Hb in the self-reference condition, the middle ones do that in the other-reference, and the right ones do that in the letter-count. The blue lines indicate timeseries oxy-Hb data of the hypoxic group, and the orange do those of the normoxic group. Across three conditions, both groups showed increase in oxy-Hb signals in response to the onset of a block, and decrease to the baseline level toward the end of the block. In the letter-count condition, both groups showed greater amplitude of oxy-Hb than those in the self- and other-reference conditions.

**FIGURE 5:**
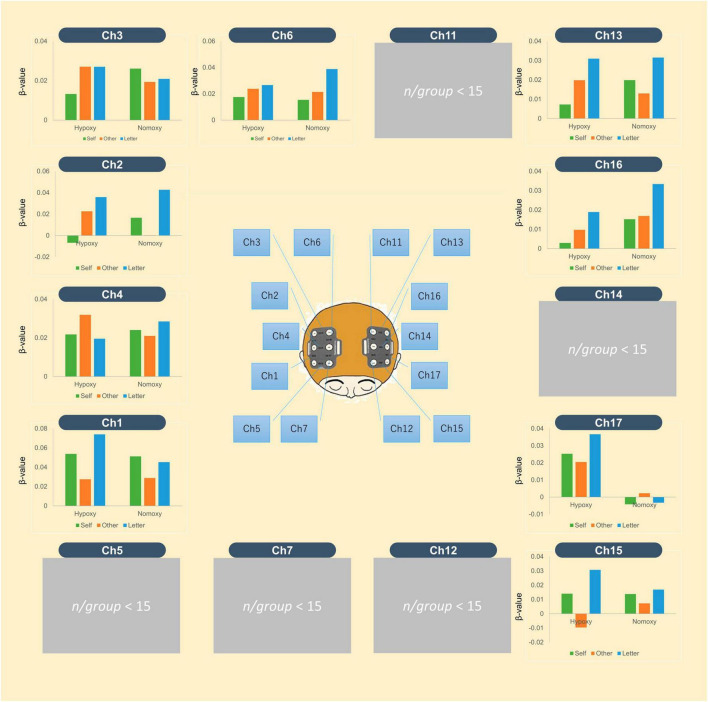
The mean beta-values of oxy-Hb signal averaged in the hypoxic and normoxic group. The middle image shows locations of 16 channels. In each bar graph, the green bar indicates mean beta-values in the self-reference condition, the orange does those in the other-reference, and blue does those in the letter-count. The left three bars belong to the hypoxic group, and the right three belong to the normoxic group.

Before performing GLMs, we inspected the averaged time-series oxy-Hb signals in each group. Figure 4 depicts the time-series oxy-Hb averages for channel 16. The oxy-Hb signals appeared to gradually increase in response to the first stimulus of a given block and remained constant until the last stimulus was presented. Such a transition in the oxy-Hb signals indicates successful measurement of cortical activation in the current cognitive task by the NIRS device. Using GLMs, we computed beta values for each task condition (i.e., self-reference, other-reference, and letter count)—summarized in Figure 5. Compared with the self- and other-reference conditions, beta values in the letter-count condition were greater in the bilateral posterior channels (Ch2, Ch11, Ch13, and Ch16) and the left frontal channel (Ch15). Similar results were obtained under both hypoxic and normoxic groups. Mixed ANOVAs revealed significant main effects of the task condition, as summarized in Supplementary Table 1, wherein *p*-values are corrected. Multiple comparisons indicated significantly greater beta values in the letter-count condition than in the self- and other-reference conditions (*p*s < 0.05) in these channels. The oxygenic level’s main effects were not observed in any of the channels. Moreover, these interactions were not statistically significant.

#### Functional connectivity analysis

**FIGURE 6:**
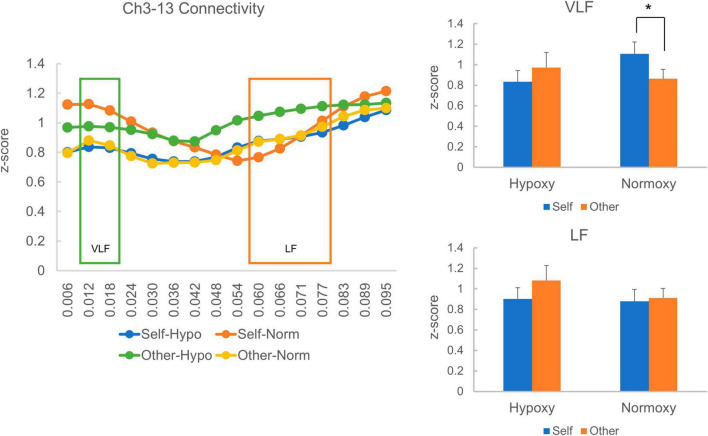
Homologous cortical functional connectivity between the channel 3 and 13. The left panel shows z-transformed magnitude of the connectivity ranged from a frequency of 0.006 to 0.095 Hz. The green box indicates magnitude of the connectivity in the very low frequency (VLF; 0.012–0.02 Hz) and the orange one indicates that in the low frequency (LF; 0.06–0.08 Hz). Each line represents z-transformed magnitude of the connectivity averaged in each reference condition (i.e., self and other) of each group (i.e., hypoxic and normoxic). The right-top panel shows z-transformed magnitude of the connectivity in the VLF. In the hypoxic group, magnitude of the VLF connectivity was equivalent between the self- and other-reference condition. In the normoxic group, by contrast, magnitude of the VLF connectivity was greater in the self-reference condition than the other-reference condition. The right-bottom panel shows z-transformed magnitude of the connectivity in the LF. Magnitude of the LF connectivity did not differ between the self- and other-reference condition in both hypoxic and normoxic groups. *p < 0.05.

In a prior study, homologous functional connectives were quantified by averaging eight homologous regions’ squared coherences. In the present study, on the contrary, we only analyzed two pairs (Ch3-13 and Ch2-16) that corresponding to the posterior and inferior temporal sites because other channels had fewer than 15 samples in each group (Ch1-17: *n*_*hypoxy*_ = 13, *n*_*normoxy*_ = 15, Ch4-14: *n*_*hypoxy*_ = 7, *n*_*normoxy*_ = 6, Ch5-15: *n*_*hypoxy*_ = 2, *n*_*normoxy*_ = 7, Ch6-11: *n*_*hypoxy*_ = 9, nnormoxy = 11, and Ch7-12: *n*_*hypoxy*_ = 4, *n*_*normoxy*_ = 6). Therefore, the present multivariate analysis is considerably primitive and further investigation is required. For the Ch3-13 pair, we had 22 and 25 samples in the hypoxic and normoxic groups, respectively. Figure 6 (left) depicts the homologous functional connectivity, which ranged from 0.006 to 0.095 Hz, indicated by the squared coherence between Ch3 and Ch13 in the self- and other-reference conditions. However, one sample in the hypoxic group was 3 *SDs* away from the squared coherence’s mean z-score, which was, consequently, removed from the statistical analysis. The green box covers the squared coherences’ magnitude in the VLF range (0.012–0.02 Hz), and the orange box represents the low-frequency (LF) range (0.06–0.08 Hz). The VLF and LF-squared coherences’ average z-scores are presented in Figure 6 (right). In the normoxic group, the VLF squared coherence was greater in the self-reference condition than in the other-reference condition. By contrast, in the hypoxic group, the VLF squared coherence was equivalent between the self- and other-reference conditions. A mixed ANOVA revealed neither a main effect of oxygenic level, *F*_(1, 44)_ = 0.33, *p* = 0.57; nor that of the task condition, *F*_(1, 44)_ = 0.34, *p* = 0.56. Notably, the ANOVA yielded a significant interaction between factors of the oxygenic level and task condition, *F*_(1, 44)_ = 4.68, *p* = 0.04. The simple main effect analysis revealed a significant main effect of the task condition in the normoxic group, *F*_(1, 24)_ = 4.71, *p* = 0.04. Regarding LF connectivity, the hypoxic and normoxic groups exhibited similar magnitudes across self- and other-reference conditions. A mixed ANOVA did not present a main effect of the oxygenic level, *F*_(1, 44)_ = 0.68, *p* = 0.42; that of the task condition, *F*_(1, 44)_ = 1.45, *p* = 0.24; or an interaction between the factors, *F*_(1, 44)_ = 0.70, *p* = 0.41.

**FIGURE 7:**
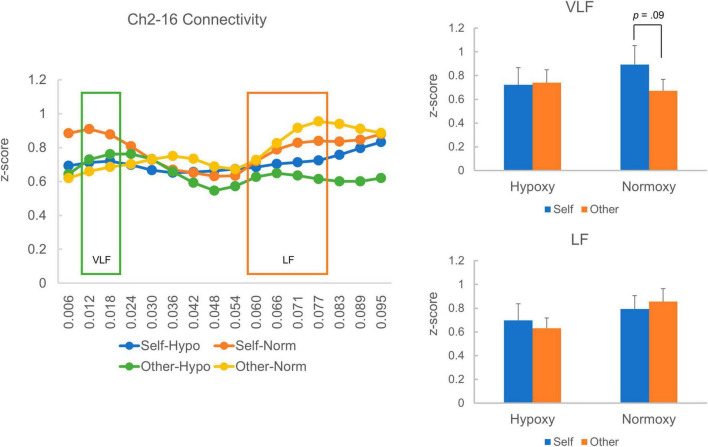
Homologous cortical functional connectivity between the channel 2 and 16. The left panel shows z-transformed magnitude of the connectivity ranged from a frequency of 0.006 to 0.095 Hz. A layout of the figural elements is identical to Figure 6. The right-top panel shows z-transformed magnitude of the connectivity in the VLF. In the hypoxic group, magnitude of the VLF connectivity was equivalent between the self- and other-reference condition. In the normoxic group, the VLF connectivity in the self-reference condition showed a trend toward a statistically greater magnitude than that in the other-reference condition (*p* = 0.09). The right-bottom panel shows z-transformed magnitude of the connectivity in the LF, which did not differ between the self- and other-reference condition in both hypoxic and normoxic groups.

Regarding the Ch2-16 pair, there were 21 and 18 samples in the hypoxic and normoxic conditions, respectively. The VLF and LF squared coherences’ average z-scores are presented in Figure 7. Like the Ch3-13 pair, the VLF coherence was numerically higher in the self-reference condition than in the other reference condition in the normoxic group (Figure 7 right-top), though a paired *t*-test indicated a trend toward a significant difference between the self- and other-reference conditions, *t* (17) = 1.78, *p* = 0.09. In the hypoxic group, VLF coherence was similar between the self- and other-reference conditions. A mixed ANOVA did not reveal a main effect of oxygen level, *F*_(1, 37)_ = 0.12, *p* = 0.73; that of the task condition, *F*_(1, 37)_ = 0.93, *p* = 0.34; or an interaction between the factors, *F*_(1, 37)_ = 1.28, *p* = 27. LF coherence did not differ between the self- and other-reference conditions in either the hypoxic or normoxic groups (Figure 7 right-bottom). A mixed ANOVA did not reveal a main effect of the oxygenic level, *F*_(1, 37)_ = 1.48, *p* = 0.23; that of the task condition, *F*_(1, 37)_ < 0.001, *p* = 0.99; or an interaction between the factors, *F*_(1, 37)_ = 0.44, *p* = 0.51.

## Discussion

The present study investigated normobaric hypoxia’s effects on the self-referential processing. Participants were randomly assigned to either normobaric hypoxic or normoxic groups, and the self-referential task was performed. Behavioral results revealed that the subsequent recognition of self-referenced words was equivalent between the two groups, whereas the recognition of other-referenced words was lower in the hypoxic group than in the normoxic group, although a statistical analysis yielded a trend toward a significant interaction. Superficial cortical activity was measured while the participants performed the task. Conventional univariate analysis did not detect any differences in neural activity between the hypoxic and normoxic groups. Interestingly, when homologous functional connectivity was analyzed, we found differences in the VLF connectivity range between the hypoxic and normoxic groups. Specifically, in the normoxic group, VLF functional connectivity was stronger in the self-reference condition than in the other-reference condition, whereas in the hypoxic group, VLF functional connectivity did not differ between self- and other-reference conditions. The absence of VLF functional connectivity reduction in the other-reference condition in the hypoxic condition may underlie the selective impairment in the other referential processing. By contrast, the self-referential process may be reserved at an oxygenic level of 13.5% because of its robust nature.

### An effect of normobaric hypoxia on the self- and non-self-referential processes

Previous studies have reported normobaric hypoxia’s exacerbating effect on a wide range of cognitive functions, including central and non-central executive functions (e.g., McMorris et al., 2017). However, few studies have investigated normobaric hypoxia’s effect on social cognition. Using the self-referential task, this study examined normobaric hypoxia’s effect on the self-referential process. Conventionally, the subsequent performance of memory retrieval is used to assess the quality of self- and other-referential processes during the reference stage (Rogers et al., 1977). During the self-referential stage, four main psychological processes are assumed to be mobilized: representation, monitoring, evaluation, and integration (Northoff and Bermpohl, 2004). In the presentation phase, participants are thought to create the representation of self-referential stimuli, which rely on the orbital-medial prefrontal cortex (Kelley et al., 2002). In the monitoring, self-referential stimuli are prioritized to be monitored due to its importance by the supragenual anterior cingulate cortex (Frith and Frith, 1999). In the evaluation, self-referential stimuli are extensively evaluated by the function of the dorsomedial prefrontal cortex (McGuire et al., 1996). In the integration phase, self-referential stimuli are concentratively associated with a personal context, which are integrated into autobiographical memory due to a contribution of the posterior cingulate cortex (Johnson et al., 2002). Those psychological processes, which depends on the cortical midline structures (CMS), enthusiastically handle self-referential stimuli, resulting in superior recognition performance relative to non-self-referential ones.

Consequently, recognition was equivalent between the hypoxic and normoxic groups in the self-reference condition. Importantly, the performance in the other-reference condition was lower in the hypoxic than in the normoxic group. The reserved performance in the self-reference condition in the hypoxic group can be interpreted as considerable engagement of the CMS, which intensively processed self-reference stimuli. According to the literature on self-pathology syndromes (Feinberg, 2011), the occurrence of self-pathology requires dysfunction at three subordinate levels—namely, cognitive deficits (level 1), self-related deficits (level 2), and deficits related to defense/adaptation/motivation (level 3). In other words, the self seems less susceptible to internal and external perturbations owing to its robust and manifold underpinnings. Both the CMS and self-pathology accounts align with the retained performance in the self-reference condition under the hypoxia.

By contrast, memory performance in the other-reference condition was lower in the hypoxic than in the normoxic group. This indicates that normobaric hypoxia impairs non-self-referential process. In terms of the depth of processing account, non-self-referential stimuli are less encoded than the self-referential one, due to its subordinate schema (Rogers et al., 1977). The organizational process account proposes that the self-referential stimuli are well recalled because self-referenced items are tightly connected each other, relative to the non-self-referenced ones (Klein and Kihlstrom, 1986). Taking those findings into consideration, the normobaric hypoxia may prevent cognitive systems from encoding or/and organizing items, resulting in selective impairment of other (i.e., Japanese former prime minister). With respect to the Feinberg’s four-tiered model on self-pathology (Feinberg, 2011), performance was retained in the self-referenced condition under the hypoxia due to multifold structure of the “self,” while performance was undermined in the other-referenced condition under the hypoxia because it only depends on the first level of the model (i.e., general cognitive system).

Performance in the letter condition was comparable between hypoxic and normoxic conditions. As the participants were only required to count the number of characters comprising an adjective, normobaric hypoxia did not seem to interfere with effortless cognitive processing. It is also possible that a floor effect may mask an effect of the hypoxia.

In sum, this study found that normobaric hypoxia impairs the non-self-referential process while secures the self-referential one. This result expands on previous studies that have found normobaric hypoxia’s interruptive effects on executive and non-executive functions (McMorris et al., 2017). Having said that, the statistical analysis yielded a trend toward a statistically significant interaction, the present interpretation needs to be cautiously evaluated.

### Effect of normobaric hypoxia on cortical activity during the self-referential task

When analyzing oxy-Hb data using a univariate method (i.e., GLM), cortical activity did not differ between the hypoxic and normoxic groups across three conditions—namely, self-reference, other-reference, and letter-count. Instead, we identified several cortical regions that presented greater activity in the letter condition than in the self- and other-reference conditions. These regions include the bilateral inferior temporal cortices and left frontal cortex. As the letter condition required participants to shift their attention to individual letters, it demanded higher visual attention. Therefore, it is plausible to assume that greater activation in the inferior temporal cortices reflects an increase in attention in the letter condition. Research on visual attention has repeatedly reported an effect of attentional modulation on inferior temporal regions (Gazzaley et al., 2005), which supports our interpretation regarding the inferior temporal activity. The left frontal cortex’s activity may reflect internal vocalization while participants counting the number of letters in the letter condition. As the left frontal channel is located near Broca’s area, which is responsible for vocalization (Jones and Fernyhough, 2007), it is reasonable to adopt an internal vocalization account.

As the self-reference task is considered to demand neural activity in the cortical midline structures (Northoff and Bermpohl, 2004), we predicted that fNIRS data’s univariate analysis would not detect differences in superficial cortical activity between self- and other-reference conditions. This prediction was, seemingly, confirmed, suggesting that univariate fNIRS analysis may be unable to capture brain activities related to the self-reference effect.

### Effect of normobaric hypoxia on homologous functional connectivity during the self- and other-referential processes

Owing to data collection problems, the homologous cortical connectivity analysis focused only on the temporal cortices (Ch3-13 and Ch2-16). Therefore, the preset results should be considered preliminary, and caution should be exercised when interpreting the results. The analysis revealed that in the normoxic group, homologous connectivity in the VLF range (0.012–0.02 Hz) was greater in the self-reference condition than in the other reference condition. By contrast, in the hypoxic group, VLF connectivity was comparable between the self- and other-reference conditions. In the low frequency range (0.06–0.08 Hz), the homologous connectivity was similar between the hypoxic and normoxic group. As homologous functional connectivity in the VLF range is proposed to reflect spontaneous neuronal activity through direct structural connections during the resting state (Sasai et al., 2011), the connectivity is highly likely to reflect certain property of the default mode network (DMN), which comprises the medial prefrontal cortex, orbital frontal cortex, lateral temporal cortex, inferior parietal lobe, posterior cingulate cortex, hippocampus, and parahippocampal cortex (Raichle, 2015). It has been repeatedly reported that regions comprising the DMN are activated during self-referential tasks, and their magnitude is stronger in the self-reference condition than in the other-reference condition (e.g., Kelley et al., 2002). It is also advocated that resting-state neural activity is closely linked to continuous self-referential processing (Northoff and Bermpohl, 2004). Therefore, greater VLF connectivity in self-reference relative to other-references in the normoxic group can be considered a normal function of the DMN. More specifically, in the self-reference condition, participants might create self-related representation, monitor self-related information, evaluate self-relatedness on the adjectives, and integrate the task experience into their autobiographical memory, recruiting the regions consisting CMS. Attenuated activity of the CMS is reported in the other-reference condition relative to the self-reference (Kelley et al., 2002; Ma and Han, 2011), which may correspond to the present result where the VLF connectivity was reduced in the other-reference condition. A similar numerical pattern of VLF connectivity—though not statistically significant—was observed in the Ch2-16 pair. When performing a paired *t*-test in the normoxic group, the VLF connectivity in the self-reference condition revealed a trend toward a greater magnitude than that in the other-reference condition (*p* = 0.09). Collectively, the greater VLF connectivity in the self-reference condition seems to reflect more involvement of the DMN regions, supporting the continuous self-referential processing.

By contrast, in the hypoxic group, the homologous cortical connectivity’s magnitude was comparable between the self- and other-reference conditions. The absence of reduced homologous connectivity in the other-reference condition relative to the self-reference condition may account for the behavioral results, whereby subsequent recognition in the other-reference condition was lower in the hypoxic than in the normoxic group. Specifically, the normobaric hypoxia disturbed semantic encoding or/and social evaluation on the other individuals. Because those psychological functions are shown to somehow depend on the CMS regions (i.e., the medial prefrontal and parietal cortices) (Baker et al., 2001; Cassidy et al., 2012), a compensatory process might arise. That is, more neural resource was allocated to the CMS regions so as to prevent semantic encoding or/and social evaluation from impairment, which unfortunately went in vain. Meanwhile, the self-referential process seems to be secured by virtue of a solid functional network within the CMS, which is less vulnerable to the hypoxia.

Homologous connectivity in the LF range was similar between the hypoxic and normoxic groups across the self- and other-reference conditions. Sasai et al. (2011) argued that a higher magnitude of frontoposterior connectivity in the LF range may reflect event-related transient coherence. This interpretation can be applied to the current results. In other words, the LF range homologous connectivity in the present study may be related to task-related activity, such as stimulus processing in the lateral cortical regions. By contrast, VLF range homologous connectivity may be linked to DMN regions’ activity.

### Limitations

Although this study employed a moderate sample size (*n* = 44 in the hypoxic group and *n* = 49 in the normoxic group), a large amount of physiological data was lost owing to technical problems. In particular, fNIRS signals were lost across several channels, thereby making it difficult to draw convincing conclusions from this study. One reason for signal loss was the use of a higher frequency (12.2 Hz) to collect the fNIRS data, which decreased the S/N ratio. We selected a higher frequency to measure homologous cortical functional connectivity following a previous study that collected data at a sampling rate of 10.0 Hz (Sasai et al., 2011). Another reason for signal loss was caused by an issue related to fixing the sensor pallet on the scalp. As the pallet was composed of plastic materials, it was less flexible to individual differences in scalp shape, skin thickness, or hair thickness. In fact, a tiny gap existed between some probes and the scalp of some participants, which made it difficult for them to emit and receive near-infrared light. Future studies consider employing flexible sensor pallets.

In the present study, we proposed a quite explorative hypothesis that magnitude of the homologous functional connectivity is associated with neural activity of the CMS, which bases the literature that found an elevated homologous functional connectivity during a resting period (Sasai et al., 2011), and that resting-state neural activity repeatedly found to be tied with continuous processing of self-referential stimuli (Northoff and Bermpohl, 2004). However, no direct evidence is yet to be obtained, which requires future studies simultaneously collecting fMRI and fNIRS data to examine its validity.

The present study examined normobaric hypoxia’s early effect on self-referential tasks; thus, an extensive effect remains to be investigated. A study of hypobaric hypoxia revealed a difference in its effect on the Simon task on days 0 and 4, wherein the reaction time was faster on day 4 than on day 0, while error rates increased on day 4 relative to day 0 (Davranche et al., 2016). Future studies are needed to test normobaric and hypobaric hypoxia’s prolonged effect on self-reference. Further studies are required to intensify the hypoxic level and test its effect on the self-referential task. At 13.5% normobaric hypoxia (approximately 3,500 m equivalent altitude), we found selective impairment in the other-reference condition that seemed to depend on basic cognitive functions. However, further hypoxia may affect the self-referential process as well. Some studies have used intensive hypoxia (e.g., 7,620 altitude) and tested its effect on executive function (Asmaro et al., 2013). Future research should consider this aspect further to elucidate whether the self-referential process is reserved in relatively intensive hypoxia.

Because it is likely that previous medical history affects brain responses in normobaric hypoxic environments, future studies need to collect comprehensive medical records and scrutinize their effects on brain responses.

## Conclusion

The present study revealed that normobaric hypoxia (oxygen concentration of 13.5%) selectively disturbed recognition of items referred to the others but not those to the self, suggesting robust nature of the self-referential processing. Univariate fNIRS did not reveal differences in brain activity between the self- and other-reference condition because fNIRS exhibits difficulty in measuring the activity of the CMS, which plays a critical role in the self-referential processing. Although the present study suffered from technical problems related to fNIRS data collection, the preliminary multivariate analysis provided a clue to a neural signature relating to selective disturbance of the other-referential processing, which involves semantic encoding or/and social evaluation, under normobaric hypoxia. Namely, it is an absence of reduced homologous cortical connectivity in the other-reference condition relative to the self-reference condition in the lateral temporal cortices. This absence may be attributable to greater need of the DMN region, which covers the CMS, to compensate for disturbances in psychological functions required for referring to non-self-individuals under the hypoxia.

## Data availability statement

The datasets for this article are not publicly available due to concerns regarding participant/patient anonymity. Requests to access the datasets should be directed to the corresponding author.

## Ethics statement

The studies involving humans were approved by The Institutional Review Board of the Faculty of Human Sciences at Shimane University. The studies were conducted in accordance with the local legislation and institutional requirements. The participants provided their written informed consent to participate in this study.

## Author contributions

TM: Writing – review and editing, Writing – original draft, Visualization, Validation, Supervision, Software, Resources, Project administration, Methodology, Investigation, Funding acquisition, Formal Analysis, Data curation, Conceptualization. NK: Writing – review and editing, Supervision, Investigation, Funding acquisition, Conceptualization. TT: Writing – review and editing, Writing – original draft, Visualization, Validation, Supervision, Project administration, Methodology, Investigation, Funding acquisition, Formal Analysis, Data curation, Conceptualization.
