# Supplementary material for: Robustness of the self-referential process under normobaric hypoxia: an fNIRS study using the GLM and homologous cortical functional connectivity analyses
Source: Front Hum Neurosci. 2024 Mar 12;18:1337798. doi: 10.3389/fnhum.2024.1337798 (PMC10967028; doi:10.3389/fnhum.2024.1337798)
Supplement: Supplementary file 1 [file Presentation_1.pptx]

## Slide 1
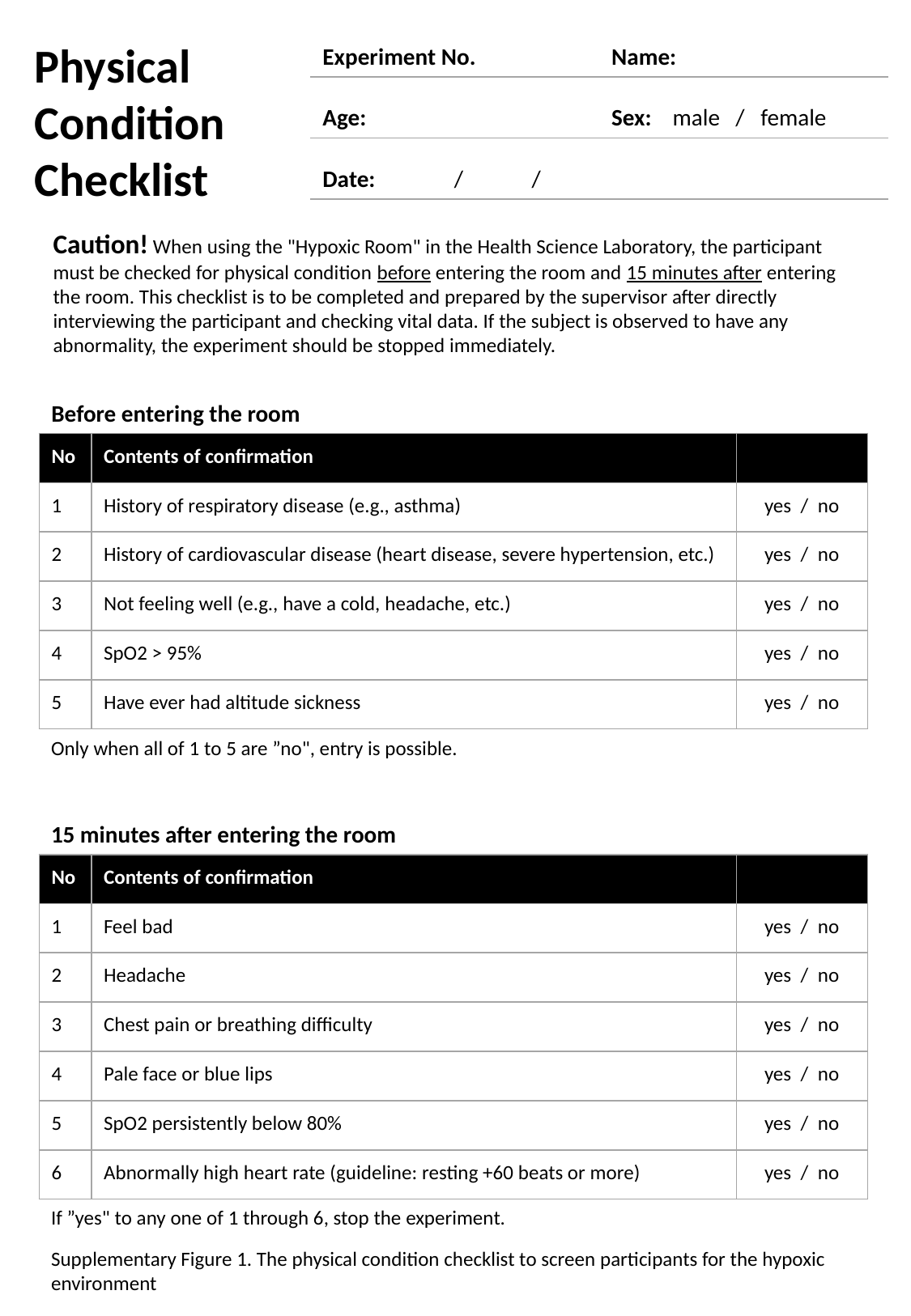

| Experiment No. | Name: |
| --- | --- |
| Age: | Sex: male / female |
| Date: / / | |
Physical
Condition
Checklist
Caution! When using the "Hypoxic Room" in the Health Science Laboratory, the participant must be checked for physical condition before entering the room and 15 minutes after entering the room. This checklist is to be completed and prepared by the supervisor after directly interviewing the participant and checking vital data. If the subject is observed to have any abnormality, the experiment should be stopped immediately.
Before entering the room
| No | Contents of confirmation | |
| --- | --- | --- |
| 1 | History of respiratory disease (e.g., asthma) | yes / no |
| 2 | History of cardiovascular disease (heart disease, severe hypertension, etc.) | yes / no |
| 3 | Not feeling well (e.g., have a cold, headache, etc.) | yes / no |
| 4 | SpO2 > 95% | yes / no |
| 5 | Have ever had altitude sickness | yes / no |
Only when all of 1 to 5 are ”no", entry is possible.
15 minutes after entering the room
| No | Contents of confirmation | |
| --- | --- | --- |
| 1 | Feel bad | yes / no |
| 2 | Headache | yes / no |
| 3 | Chest pain or breathing difficulty | yes / no |
| 4 | Pale face or blue lips | yes / no |
| 5 | SpO2 persistently below 80% | yes / no |
| 6 | Abnormally high heart rate (guideline: resting +60 beats or more) | yes / no |
If ”yes" to any one of 1 through 6, stop the experiment.
Supplementary Figure 1. The physical condition checklist to screen participants for the hypoxic environment
