# Supplementary material for: Robustness of the self-referential process under normobaric hypoxia: an fNIRS study using the GLM and homologous cortical functional connectivity analyses
Source: Front Hum Neurosci. 2024 Mar 12;18:1337798. doi: 10.3389/fnhum.2024.1337798 (PMC10967028; doi:10.3389/fnhum.2024.1337798)
Supplement: Supplementary file 2 [file Presentation_2.pdf]

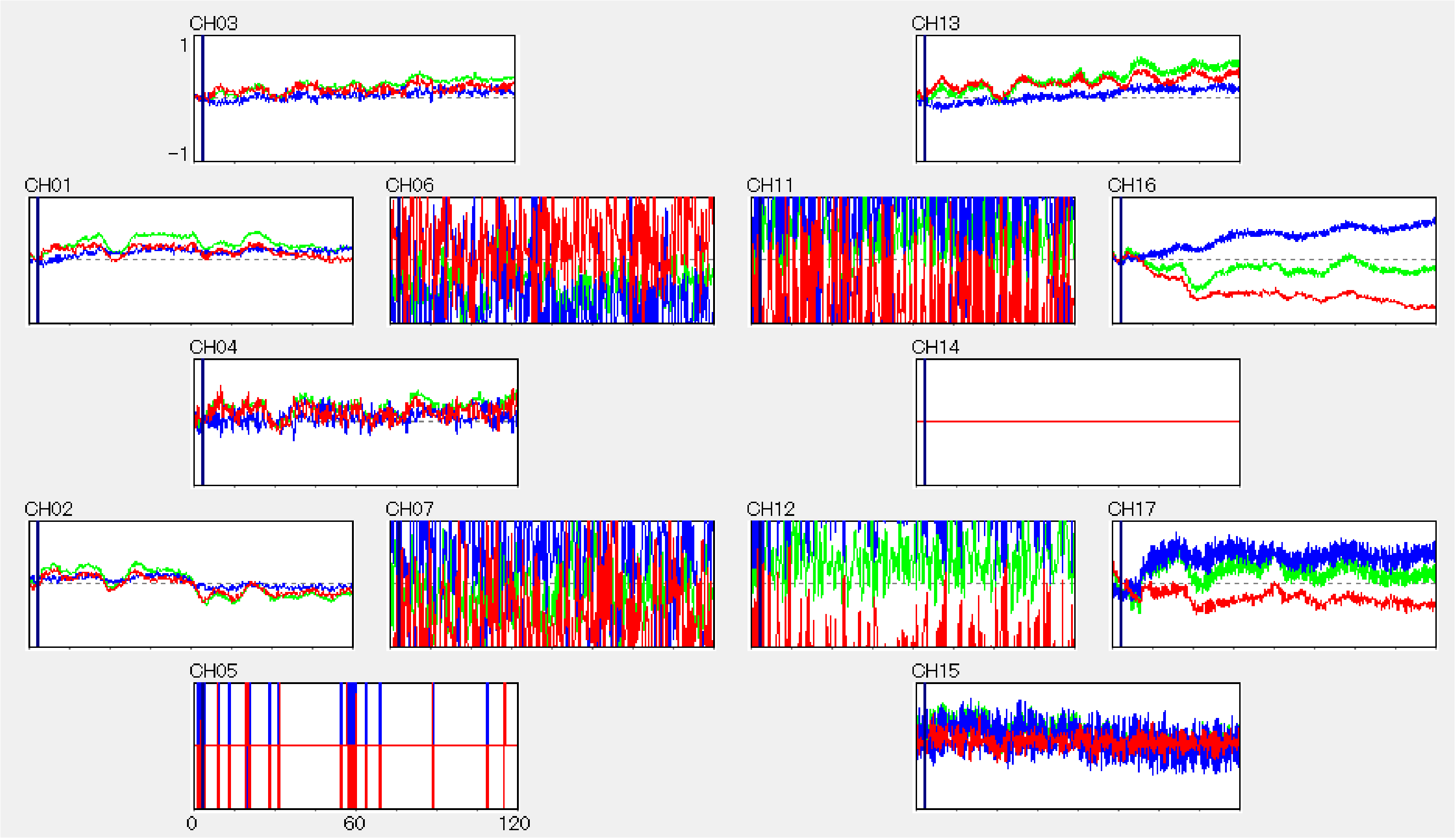

Supplementary Figure 2. Sample fNIRS timeseries data of one participant. The timeseries data covers fNIRS signal from the beginning of the self-reference task to the end. In channels 5, 6, 7, 12, and 15, fNIRS data were far apart from the predicted signal. In channel 14, timeseries data were completely lost. The redline represents oxy-Hb, the blueline deoxy-Hb, and the green total-Hb.

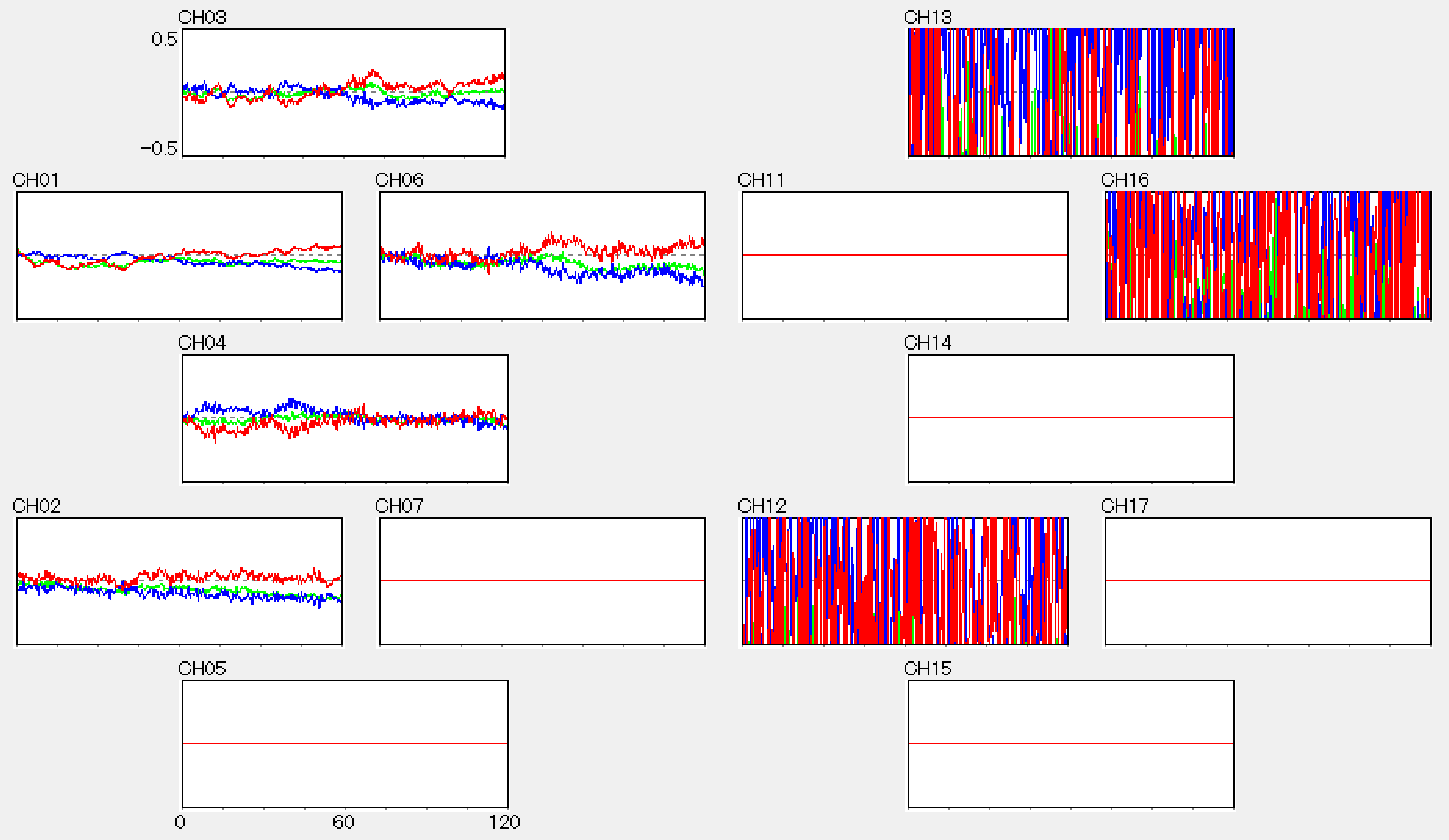

Supplementary Figure 3. Sample fNIRS timeseries data of another participant. In this subject, timeseries data were completely lost in 5 channels (Ch5, 7, 11, 14, 15, and 17). In addition, three channels (Ch12, 13, and 16) contained tremendous artifact. The redline represents oxy-Hb, the blueline deoxy-Hb, and the green total-Hb.
